# An exploration of the barriers to attendance at the English Stop Smoking Services

**DOI:** 10.1016/j.abrep.2018.10.005

**Published:** 2018-11-01

**Authors:** Dimitra Kale, Hazel Gilbert, Stephen Sutton

**Affiliations:** a Research Department of Primary Care and Population Health, UCL, London, UK; b Institute of Public Health, University of Cambridge, Cambridge, UK

**Keywords:** Smoking cessation, Stop smoking service, Treatment access

## Abstract

**Introduction:**

Despite the availability of effective stop smoking assistance, most smokers do not utilise formal cessation programmes such as the English Stop Smoking Services (SSS). We modified the Treatment Barriers Questionnaire (TBQ), developed in the USA, and distributed it to a sample of English smokers to explore the most important barriers to the use of the SSS.

**Methods:**

Participants of Start2quit, a randomised controlled trial aiming to increase attendance at the SSS using tailored risk information and ‘taster’ sessions, who reported at follow-up that they had not attended the SSS, were asked to complete the TBQ; 672 (76.9% response rate) were retained for analysis. Principal Component Analysis (PCA) was conducted to examine the structure of the data. Multiple linear regressions were used to determine whether any participant characteristics were associated with particular barriers.

**Results:**

The most commonly endorsed items related to a lack of information on and a lack of confidence in the efficacy of the SSS. PCA yielded seven factors: Work and time constraints (Factor1); Smokers should quit on their own (Factor2); Nothing can help in quitting smoking(Factor3); Disinterest in quitting (Factor4); Lack of social support to attend (Factor5); Lack of privacy at programmes (Factor6); Lack of information and perceived availability (Factor7). Age was associated with Factors 1, 3 and 4, motivation to quit with Factors 2 and 4, and confidence in quitting with Factors 1, 2, and 3.

**Conclusions:**

The findings suggest that many barriers exist, and they vary according to smoker demographics and characteristics, pointing to the need for tailored recruitment strategies.

**Trial registration:**

ISRCTN76561916.

## Highlights

• Identifying barriers to the use of stop smoking services
• The findings suggest that many barriers exist.
• Smokers lack information about stop smoking services.
• Barriers vary according to smoker demographics and characteristics.

## 1 Introduction

Smoking is one of the leading causes of morbidity, mortality and health inequalities globally (World Health Organization, 2013). Research aimed at tackling this problem has found that structured behavioural support in combination with pharmacotherapy is more effective way of helping people to achieve prolonged smoking abstinence than attempting to quit without help (Stead & Lancaster, 2012). In many countries, stop smoking interventions have been developed to offer intensive advice and support to smokers motivated to quit (World Health Organization, 2013). Evidence also indicates that smokers who take advantage of these stop smoking programmes have a greater chance of stopping smoking and remaining abstinent than those who try to quit on their own (West & Stapleton, 2008; Zhu, Melcer, Sun, Rosbrook, & Pierce, 2000). Despite the availability of this effective assistance in quitting, the majority of smokers do not utilise any formal cessation programmes (Fiore et al., 1990; Hughes, Marcy, & Naud, 2009; Lichtenstein & Glasgow, 1992; Owen & Davies, 1990). Instead, approximately half of smokers make an independent, unassisted quit attempt every year with <5% of such attempts being maintained a year later (Cokkinides, Ward, Jemal, & Thun, 2005; Edwards, Bondy, Callaghan, & Mann, 2014; Hughes, Keely, & Naud, 2004).

In 1999 the Department of Health in UK set up the English Stop Smoking Services (SSS), a national network of specialist smoking cessation services, offering free intensive advice and support to smokers wanting to quit, in group or one-to-one sessions. However, the proportion of smokers in England using the SSS has always been low at <5% (West & Brown, 2012), and the latest figures show a continuing downward trend (Health and Social Care Information Centre, 2015). Thus research is needed to assess in more detail the barriers to the use of the SSS, to explain this low attendance, and to enable the development of more focused promotion of treatment services.

The importance of further investigation into these barriers to the uptake of intensive treatment programmes was highlighted by Fiore (Fiore et al., 2008). Research both before and since this call has suggested several factors that may account for the low rates of attendance at smoking cessation programmes, including a lack of awareness of the services available, low expectations about their effectiveness and misunderstanding of how treatments work (Hammond, McDonald, Fong, & Borland, 2004; Hughes et al., 2009; Roddy, Antoniak, Britton, Molyneux, & Lewis, 2006; Smith, Carter, Chapman, Dunlop, & Freeman, 2015). Smokers report having insufficient knowledge of the available services, and this lack of knowledge can lead to smokers underestimating the benefits of treatment and to the belief that the service would not help them (Christiansen, Reeder, Terbeek, Fiore, & Baker, 2015; Hammond et al., 2004; Hughes et al., 2009; Paul et al., 2004; Roddy et al., 2006; van Rossem et al., 2015; Vogt, Hall, & Marteau, 2010). Many smokers also believe that motivation and willpower is sufficient. The belief that ‘if you really want to quit smoking you will succeed on your own as well as you would with help’ is prevalent (Hammond et al., 2004). Concern about lack of empathy from health professionals and notions that treatments are unavailable and hard to access also permeate the literature (Christiansen et al., 2015; Hughes et al., 2009; Roddy et al., 2006; Smith et al., 2015; Vogt et al., 2010).

Copeland and colleagues adopted a more structured approach to assess the barriers to attending smoking cessation programs specifically in low socio-economic smokers in the USA. Using the Treatment Barriers Questionnaire (TBQ) with this group, a validated 40-item measure of reasons for not entering smoking cessation programmes empirically derived from existing literature (Copeland et al., 2010), they conducted an exploratory Principal Components Analysis (PCA) identifying seven factors: Preparedness to quit smoking; Work and time constraints; Smokers should quit on own; Opinions about professional assistance; Mobility limitations; Misinformation about professional assistance; and Insurance limitations. These barriers largely map on to those identified in the literature, but emphasise financial and mobility issues more common in low socio-economic groups. Copeland then conducted regression analyses to determine whether these factors related to characteristics such as demographics, stage of readiness to quit smoking and dependence.

The present study is based on existing findings and many of these findings are consistent with theories and models that have been used to understand the uptake of services, such as the Health belief model (Glanz, Rimer, & Lewis, 2002). We modified the TBQ and distributed it to a sample of English smokers to 1) identify the most important barriers perceived by smokers that prevent them from seeking help to quit, specifically to attending the SSS; 2) to explore, without prior hypothesis, the factor structure of the adapted TBQ in a sample of English smokers; and 3) to determine whether these barriers differ according to demographics, smoker characteristics, attitudes and beliefs about smoking and smoking cessation, social-environmental factors and previous attendance at the SSS.

## 2 Methods

Data for the present study were collected as part of the Start2quit study, a randomised controlled trial aiming to increase the number of people attending the SSS using personalised tailored risk information and introductory ‘taster’ sessions (Gilbert et al., 2012).

### 2.1 Start2quit study procedure

The study was approved by the London-Surrey Borders Research Ethics Committee (Reference number 10/H0806/20). Eighteen SSSs across England and 99 general practices within the SSS areas were recruited into the trial between February 2011 and October 2013. All current cigarette and roll-up smokers over the age of 16 were identified from their medical records in participating practices (*n* = 106,819) and were sent an invitation to participate in the study by their GP along with a Smoking Behaviour Questionnaire. Smokers returning the questionnaire and giving consent (*n* = 4384) were randomly allocated to the control group (*n* = 1748) or to the intervention group (*n* = 2636). Those in the control group were sent a generic letter advertising the SSS in their area, and those in the intervention group were sent a computer-tailored letter signed by their GP containing personalised risk information and an invitation to a no-commitment ‘taster session’ to find out more about the SSS. All participants were contacted 6 months after the date of randomization, by telephone, to assess their attendance at the SSS and current smoking status. Participants unable to be contacted or unable to complete a telephone interview were sent a postal questionnaire. If a participant did not wish to complete the telephone interview or paper questionnaire, the interviewer attempted to ask four basic questions relevant to the primary and main secondary outcome. In total 2910 (66.4%) completed the full telephone interview, 302 (6.9%) completed a shorter postal questionnaire, and 160 (3.7%) completed the four basic questions, giving a total response rate of 76.9% (3372). There was no difference in follow-up response between the treatment groups, 76.7% vs. 77.3% in the intervention and control groups respectively. More details about randomization and follow-up procedures can be found elsewhere (Gilbert et al., 2012).

Results from the Start2quit trial showed that this intervention more than doubled the odds of attendance at the SSS (17.4 vs 9.0%, OR 2.12 [1.75–2.57], *p* < 0.001). The number completing the 6-week SSS course was significantly higher in the intervention group (14.5 vs 7.0%, OR 2.24 [1.81–2.78], *p* < 0.001), and 7-day point-prevalent abstinence at the 6-month follow-up, validated by salivary cotinine, was significantly increased (9.0 vs 5.6%, OR 1.68 [1.32–2.15], *p* < 0.001, (Gilbert et al., 2017).

### 2.2 Participants

Participants in the Start2quit study who completed the 6-month follow-up interview by telephone and reported not attending the SSS during the 6-month follow-up period were asked to complete a further postal questionnaire. Those who agreed (*n* = 1597) were mailed the TBQ to complete and return in a postage paid envelope. Participants who did not reply after two weeks were sent a duplicate TBQ. A total of 758 completed TBQs were returned, a response rate of 47.5%. On examination it was found that 65 had attended a taster session, 11 had attended the SSSs and 10 had attended both a taster session and the SSSs. These people were excluded so that the final sample (*n* = 672) consisted only of smokers who had not attended either a taster session or a course run by the SSS in the previous 6 months.

### 2.3 Measures

Participant characteristics were assessed at baseline: demographics (age, gender, marital status, ethnicity, and education), social deprivation (Index of Multiple Deprivation (IMD) score, the UK Government's official measure of multiple deprivation at small area level (Noble et al., 2007)) and social-environmental measures (living with other smokers), smoking history (age starting smoking, previous quit attempts), nicotine dependence (measured by the number of cigarettes smoked daily and time from waking to first cigarette), intention to quit, and motivation, determination and confidence to quit (all rated on a five-point Likert-type scale, 1 = not all to 5 = extremely). Previous attendance at the SSS and self-reported health problems associated with smoking were also assessed.

The TBQ used in this study was adapted from the 40-item questionnaire developed and validated in the USA (Copeland et al., 2010). Items that did not apply to an English population (e.g. insurance coverage) were removed and British English spelling was used throughout. The final TBQ, comprising 36 items, was piloted with SSS managers to test for appropriateness to the service. Participants were asked to rate possible reasons for not entering a SSS programme (structured group or one-to-one therapy for smokers led by professionals e.g. stop smoking advisor, practice nurse or health care assistant) using a 5-point Likert scale (1 = strongly disagree to 5 = strongly agree).

### 2.4 Analysis

Descriptive statistics were used to describe the sample characteristics. Chi-square or *t*-tests as appropriate were used to compare responders and non-responders to the TBQ and also to compare intervention and control group participants. The proportions endorsing each item and the number of missing values per item were calculated.

Exploratory factor analysis using principal component analysis (PCA) was conducted to examine the factor structure of the TBQ on a sample of English smokers. Varimax rotation was used. Oblique rotation was also performed, but this produced a similar solution. To determine the number of factors to retain, the scree criterion (Cattell, 1966) was applied. Items with factor loadings of at least 0.40 and with no cross-loadings were retained for rotation. Upon ascertaining the TBQ's factor structure, inter-item reliability (coefficient alpha) was determined for each of the factors. The effect on alpha of deleting each item was examined, and any item that did not appear to improve reliability substantially was eliminated. The mean score of the remaining items that loaded on each factor was calculated to construct a scale for each factor, and Pearson's correlations were used to examine their inter-relationships.

Multiple linear regressions were used to determine whether any participant characteristics were associated with particular barriers to entering the SSS. Separate regressions were performed for each scale produced by PCA. Although some of the variables were not normally distributed, multiple linear regression is robust to departures from normality. None of the variance inflation factors for the predictor variables in any regressions analyses exceeded four, suggesting that multicollinearity is not a problem. Moreover, the regression analyses were repeated to examine the effect of adjusting for clustering by SSS by using the ‘cluster’ option in STATA. The results were broadly similar to the unadjusted results, therefore we report only the unadjusted results.

In order to control for error due to the number of comparisons being made, we adopted a more conservative level of significance. Therefore for all analyses a variable was considered to be significantly associated with the outcome if the *p*-value was <0.01 (Chen, Feng, & Xiaolian, 2017).

## 3 Results

### 3.1 Participants characteristics

Demographic and smoking characteristics are shown in Table 1. Although participants were randomised 2:1 to the intervention and control groups respectively (Gilbert et al., 2012), fewer participants in the control group attended the SSS, thus slightly more of the respondents to the TBQ were in the control group (53.6%).

**Table 1:** Demographic and smoking characteristics of respondents.

	Respondents (*n* = 672)
	*n*(%)/mean(SD)
Intervention (%)	312(46.4)
Demographics	
Male (%)	330(49.1)
Mean age (years) (SD)	51.4(12.9)
Married/living with spouse (%)	399(59.4)
Highest qualification GCE – A-level^a^ or higher (%)	255(39.0)
Ethnic background White (%)	664(99.3)
Deprivation (IMD score)^b^ (%)	
Quintile 1 (least deprived)	95(14.2)
Quintile 2	98(14.6)
Quintile 3	160(23.9)
Quintile 4	162(24.2)
Quintile 5 (most deprived)	154(23.0)
Mean IMD score (SD)	23.52(14.28)
Live with smokers (%)	220(32.8)
Smoking characteristics	
Nicotine Dependence score (0–6)^c^ (%)	
Low (score = 0–2)	300(45.1)
Medium (score = 3)	207(31.1)
High (score = 4–6)	158(23.8)
Mean (SD)	2.5(1.5)
Age started smoking (%)	
<14	103(15.3)
14–16	337(50.1)
>16	232(34.5)
Intention and motivation to quit	
When planning to quit (%)	
In next 2 weeks	91(14.2)
Next 30 days	107(16.7)
Next 6 months	310(48.4)
Not within 6 months	133(20.7)
Longest previous quit attempt (%)	
<24 h	48(7.2)
1–6 days	96(14.4)
1–4 weeks	104(15.6)
>1 month	419(62.8)
Previous attended SSS (%)	235(35.0)
Mean score (SD) ‘How much do you want to quit?’ (1 = not at all, 5 = extremely)	3.56(0.93)
Mean score (SD) ‘How determined are you to quit?’ (1 = not at all, 5 = extremely)	3.54(0.98)
Mean score (SD) ‘How confident are you that you can quit?’ (1 = not at all, 5 = extremely)	2.57(1.06)
Health	
Health problems (self-reported) (%)	160(24.5)

a Compulsory school in the UK ends at age 16. A levels or equivalent qualifications are generally taken in the last year of school at age 18 and are prerequisites for university entrance.

b Index of Multiple Deprivation score is the Government's official measure of multiple deprivation at small area level.

c Dependence score was computed from the number of cigarettes per day and time from waking to first cigarette and is a score between 0 and 6 categorised as low (0–2) medium(3) and high (4–6).

There were a number of differences between responders and non-responders to the TBQ. Responders were significantly older (51.4 vs 48.5 years, *p* < 0.001) and more likely to have higher educational qualifications (A level or post school)(39% vs 29.7%, *p* < 0.001). They were less likely to be intending to quit in the next 30 days (30.9% vs 40.8%, *p* < 0.001), and were less motivated (3.56 vs. 3.7, *p* = 0.003) and less determined to quit (3.54 vs. 3.72, *p* < 0.001) than non-responders. They were also more likely to have quit in the past for longer than one month (62.8% vs 53.5%, *p* = 0.002).

Differences between the intervention and control group respondents were minimal, the only significant ones being age (48.9 vs 53.5, *p* < 0.001) and previous SSS attendance (29.2% vs 40.0%, *p* = 003).

### 3.2 TBQ item endorsement

The proportion endorsing each item is presented in Table 2. Of the 672 responses to the TBQ, 489 answered all 36 survey items, while 183 had one or several missing responses. On average 3.2 of responses per item (range from 0.2% to 15.9%) were missing.

**Table 2:** TBQ: Missing items and proportion of respondents endorsing each item.

Item	Missing values *n* (%)	Agree/Strongly agree *n* (%)
Q1 Nicotine replacement therapy is too expensive	9(1.3)	340(50.6)
Q2 I don't have the time to commit to a programme	6(0.9)	230(34.2)
Q3 Most programmes are conducted in groups and I'm not comfortable meeting in a group	18(2.7)	278(41.4)
Q4 I don't want to give up smoking	4(0.6)	88(13.1)
Q5 People shouldn't need help in quitting smoking	18(2.7)	76(11.3)
Q6 I should be able to quit on my own	3(0.4)	320(47.6)
Q7 I think nicotine replacement therapy (e.g., the nicotine patch, gum) alone will be effective	9(1.3)	127(18.9)
Q8 I don't think I can quit smoking, regardless of what I do	3(0.4)	113(16.8)
Q9 I've been through programmes in the past, and they didn't help me quit smoking	56(8.3)	170(25.3)
Q10 I'm young and healthy and don't need to quit right now	29(4.3)	16(2.4)
Q11 I plan to quit on my own soon	21(3.1)	243(36.2)
Q12 My work schedule is too hectic	23(3.4)	217(32.3)
Q13 There is nobody who could watch my children	82(12.2)	31(4.6)
Q14 I can't afford to spend my time that way	29(4.3)	112(16.7)
Q15 I like smoking and don't want to give it up	16(2.4)	117(17.4)
Q16 There is no service near my home	23(3.4)	48(7.1)
Q17 I am not aware of any programmes in this area	14(2.1)	142(21.1)
Q18 My spouse/partner smokes and I wouldn't want to quit without him/her	58(8.6)	65(9.7)
Q19 I will just end smoking again	11(1.6)	159(23.7)
Q20 I have a health problem that would prevent me from attending a programme	14(2.1)	40(6.0)
Q21 There is no point in quitting, the damage has been done to my health	16(2.4)	59(8.8)
Q22 I don't think smoking is really that bad for me	8(1.2)	25(3.7)
Q23 I can't afford childcare	107(15.9)	34(5.1)
Q24 My work schedule would prevent me from attending a regularly scheduled programme	40(6.0)	225(33.5)
Q25 I've tried quitting smoking in the past, and just couldn't do it.	20(3.0)	361(53.7)
Q26 I can quit whenever I want to on my own	6(0.9)	105(15.6)
Q27 I have no way of getting to the meetings	33(4.9)	68(10.1)
Q28 Any smoker can quit on his/her own if he/she puts his/her mind to it	1(0.1)	237(35.3)
Q29 My health problems prevent me from getting out	20(3.0)	40(6.0)
Q30 I'll just hear things I've heard over and over again about smoking	7(1.0)	315(46.9)
Q31 I won't learn anything new and helpful	15(2.2)	185(27.5)
Q32 I don't know much about what programmes do to help smokers quit	6(0.9)	307(45.7)
Q33 I would need more information on specific programmes to make a decision whether I would attend	13(1.9)	343(51.0)
Q34 Those programmes are too time-consuming	10(1.5)	128(19.0)
Q35 I wouldn't want to talk about my smoking with total strangers	9(1.3)	166(24.7)
Q36 Most smokers don't need that kind of help to quit smoking	5(0.7)	61(9.1)

The most commonly endorsed item was ‘I've tried quitting smoking in the past, and just couldn't do it’, followed by ‘I would need more information on specific programmes to make a decision whether I would attend‘. The item ‘nicotine replacement is too expensive’ was endorsed by 50.6% of the sample. Other highly endorsed items also related to a lack of information on and a lack of confidence in the efficacy of the SSS (items Q6, Q30 and Q32). More intervention than control group participants endorsed ‘There is no service near my home’ and ‘I've been through programmes in the past and they didn't help me give up smoking’, perhaps accounted for by the significantly greater proportion of the control group who had attended the SSS in the past.

### 3.3 Principal component analysis

The PCA with varimax rotation yielded seven factors as the most interpretable, explaining 52.9% of the total variance. Thirty items were retained and the scales were named for their item content: 1) Work and time constraints (5 items, mean = 2.70, SD = 0.92); 2) Smokers should quit on their own (4 items, mean = 2.84, SD = 0.78); 3) Nothing can help in quitting smoking (5 items, mean = 2.91, SD = 0.76); 4) Disinterest in quitting (4 items, mean = 1.98, SD = 0.68); 5) Lack of social support to attend SSS (6 items, mean = 1.76, SD = 0.60); 6) Lack of privacy at SSS programmes (2 items, mean = 2.88, SD = 1.03); 7) Lack of information on and perceived availability of SSS (4 items, mean = 2.77, SD = 0.73). Coefficient alpha ranged from 0.66 to 0.84 across the seven scales. Table 3 displays the factor structure of the TBQ. Interscale correlations of the TBQ are shown in Table 4.

**Table 3:** TBQ: scales, items and factor loadings.

Scale (coefficient alpha reliability, total variance explained)	Loading
1. Work and time constraints (α = 0.84, 13.90% of total variance, mean = 2.70, SD = 0.92)	
My work schedule would prevent me from attending a regularly scheduled programme	0.850
My work schedule is too hectic	0.841
I don't have the time to commit to a programme	0.793
I can't afford to spend my time that way	0.720
Those programmes are too time-consuming	0.600
2. Smokers should quit on their own (α = 0.67, 10.18% of total variance, mean = 2.84, SD = 0.78)	
I should be able to quit on my own	0.732
Any smoker can quit on his/her own if he/she puts his/her mind to it	0.723
I plan to quit on my own soon	0.582
People shouldn't need help in quitting smoking	0.560
3. Nothing can help in quitting smoking (α = 0.73, 8.50% of total variance, mean = 2.91, SD = 0.76)	
I've tried quitting smoking in the past, and just couldn't do it	0.671
I don't think I can quit smoking, regardless of what I do	0.652
I will end up smoking again	0.630
I'll just hear things I've heard over and over again about smoking	0.578
I won't learn anything new and helpful	0.549
4. Disinterest in quitting (α = 0.70, 6.46% of total variance, mean = 1.98, SD = 0.68)	
I don't want to give up smoking	0.734
I like smoking and don't want to give up	0.697
I don't think smoking is really that bad for me	0.659
I'm young and healthy and don't need to quit right now	0.621
5. Lack of social support to attend SSS (α = 0.71, 5.28% of total variance, mean = 1.76, SD = 0.60)	
I can't afford childcare	0.774
There is nobody who could watch my children	0.700
My health problems prevent me from getting out	0.638
I have a health problem that would prevent me from attending a programme	0.609
I have no way of getting to the meetings	0.421
My spouse/partner smokes and I wouldn't want to quit without him/her	0.408
6. Lack of privacy at SSS programmes (α = 0.70, 4.68% of total variance, mean = 2.88, SD = 1.03)	
I wouldn't want to talk about my smoking with total strangers	0.771
Most programmes are conducted in groups and I am not comfortable meeting in a group	0.764
7. Lack of information on and perceived availability of SSS (α = 0.66, 3.91% of total variance, mean = 2.77, SD = 0.73)	
I am not aware of any programmes in this area	0.705
I don't know much about what programmes do to help smokers quit	0.687
There is no service near my home	0.644
I would need more information on specific programmes to make a decision whether I would attend	0.618

**Table 4:** Pearson's correlations between TBQ scales.

Scale	1	2	3	4	5	6	7
1 Work and time constraints	–	0.15	0.14	0.05	0.25	0.20	0.19
2 Smokers should quit on their own		–	−0.06	0.01	0.01	0.12	−0.02
3 Nothing can help in quitting smoking			–	0.42	0.20	0.23	0.13
4 Disinterest in quitting				–	0.14	0.05	0.01
5 Lack of social support to attend SSS					–	0.19	0.25
6 Lack of privacy at SSS programmes						–	0.09
7 Lack of information on and perceived availability of SSS							–

### 3.4 Association between barriers and demographic and smoking characteristics

Multiple regression analyses showed that different predictors were significant for each factor (Table 5).

**Table 5:** Associations between barriers and demographic and smoking characteristics.

	Factor 1	Factor 2	Factor 3	Factor 4	Factor 5	Factor 6	Factor 7
Variable	Beta	Beta	Beta	Beta	Beta	Beta	Beta
Demographic variables							
Age	−0.29⁎⁎	0.03	0.13⁎	0.18⁎⁎	0.03	−0.02	−0.10
Gender	−0.02	−0.08	0.06	−0.02	−0.02	0.08	0.01
Marital status	0.05	0.03	−0.01	−0.02	−0.02	−0.04	0.06
Social deprivation	−0.05	−0.01	−0.03	0.002	0.14⁎	0.08	−0.02
Education	−0.02	0.07	0.02	−0.03	−0.06	−0.15⁎	0.04
Living with other smokers	−0.05	−0.05	0	0.04	0.13⁎	−0.01	0.05

Measures of smoking history and nicotine dependence							
Nicotine dependence index	0.01	−0.13⁎	0.05	0.04	0.05	0.06	0.003
Age started smoking	0.04	0.03	−0.06	−0.03	−0.10	0.07	−0.07

Intention and motivation to quit							
Intention to quit	−0.05	−0.03	−0.04	0.06	−0.08	0.17⁎	0.02
Previous quit smoking for 3 months or more	0.06	−0.01	−0.06	−0.01	−0.02	−0.07	0.02
Previous attended SSS	−0.05	−0.07	0.07	−0.002	0.01	−0.06	−0.16⁎
Motivation to quit	−0.08	−0.15⁎	0.03	−0.34⁎	0.04	−0.04	0.10
Confidence in quitting	−0.18⁎⁎	0.18⁎⁎	−0.40⁎	0.06	−0.06	−0.01	0.02

Health							
Health problems	−0.13⁎	−0.05	0.11	−0.03	−0.01	0.06	0.03

Beta = Beta standardized coefficients.

⁎ *p* < 0.01.

⁎⁎ *p* < 0.001.

Endorsement of Factor 1, Work and time constraints, was significantly predicted by younger age (β = −0.29, *p* < 0.001), and having lower confidence in quitting (β = −0.18, *p* = 0.001). Having no health problems related to smoking (β = −0.13, *p* = 0.009) also predicted endorsement of this factor.

Factor 2, Smokers should quit on their own, was associated with having higher confidence in quitting (β = 0.18, *p* = 0.001). Lower nicotine dependence (β = −0.13, *p* = 0.01) and lower levels of motivation to quit were also associated with this factor (β = −0.15, *p* = 0.018).

Those with lower confidence in quitting (β = −0.4, *p* < 0.001) and older smokers (β = 0.13, *p* = 0.008) were significantly more likely to endorse Factor 3, Nothing can help in quitting smoking.

Factor 4, Disinterest in quitting, was also endorsed by older smokers (β = 0.18, *p* < 0.001), as well as by those with lower motivation (β = −0.34, *p* < 0.001).

Social deprivation (β = 0.14, *p* = 0.004) and living with other smokers (β = 0.13, *p* = 0.012) were associated with Factor 5, Lack of social support to attend the SSS.

Factor 6, Lack of privacy at SSS programmes, was significantly associated with higher intention to quit (β = 0.17, *p* = 0.003) and lower educational level (β = −0.15, *p* = 0.003).

Finally, those who had not attended the SSS in the past were more likely to endorse Factor 7, Lack of information and perceived availability (β = −0.16, *p* = 0.002).

## 4 Discussion

This study identified barriers to entering the SSS in a sample of smokers recruited from general practices in England. The most common barriers, indicated by the highest proportion of endorsements, suggested that smokers either lack information about the SSS or do not have confidence in the efficacy of the service. Furthermore, the prevalent belief that help is not needed was confirmed in this sample.

Exploratory PCA of the TBQ yielded a 30-item, seven-factor solution accounting for 52.9% of the variance. Work and time constraints accounted for the highest proportion of variance (13.9%); the notion that smokers should quit on their own and that nothing can help in quitting smoking were the next highest (10.2% and 8.5% respectively). Other barriers emerging were disinterest in quitting, lack of social support to attend, lack of privacy at SSS programmes, and a lack of information about, and perceived availability of the SSS.

The results of the PCA in the present study confirm a structure comparable to the original US questionnaire (Copeland et al., 2010) comprising similar scales. However in the present study some scales were renamed to be more appropriate. The factors ‘Work and time constraints’ and ‘Smokers should quit on their own’ correspond to those found by Copeland and colleagues. ‘Preparedness to quit’ equates to ‘Disinterest in quitting’ and represents smokers not ready to quit. Copeland's scales ‘Opinions about professional assistance’ and ‘Misinformation about professional assistance’ correspond to the combined and renamed ‘Nothing can help in quitting smoking’, a factor that is more specific in representing the belief that support offered is ineffective. Copeland's ‘Misinformation’ factor includes items that could also be included in the factor ‘Lack of information and perceived availability’, and ‘Mobility’ largely equates to ‘Lack of social support’. The highest rated items largely map onto these factors. While one of the highest rated items ‘nicotine replacement therapy is too expensive’ did not load on any factor, this also suggests a lack of sufficient information about the service and expectations of the treatment.

The mean scores for each subscale indicated the belief that ‘Nothing can help in quitting’ was an important barrier to attendance at the SSS. Items loading on this factor were more likely to be endorsed by older smokers and those with lower levels of confidence in quitting. Also highly rated was the belief that wanting to quit is sufficient and smokers should quit on their own. Smokers with higher confidence in quitting as well as participants with lower nicotine dependence scores were more likely to believe that smokers do not need professional help to quit. While research suggests that support for smoking cessation is used primarily by the most addicted group of smokers (Balmford & Borland, 2008; Hammond et al., 2004; Hughes et al., 2009; Kotz, Fidler, & West, 2009), the relationship between self-efficacy and abstinence is far from clear, and has not been found to consistently predict the success of an attempt (Gwaltney, Metrik, Kahler, & Shiffman, 2009; Vangeli, Stapleton, Smit, Borland, & West, 2011). Hence the belief that high confidence in quitting negates the need for support is a misconception in need of correction.

High ratings on lack of privacy at the SSS and lack of information and perceived availability of the SSS indicate that these are also important barriers to attendance. The privacy issue was associated with lower educational level and greater intention to quit, while lack of information was highly endorsed by those who had not attended a smoking cessation programme in the past.

‘Work and time constraints’ appears to be a key barrier to attendance for younger smokers and those with no health problems related to smoking. Citing work and time constraints as a barrier suggests an interest in quitting if time permitted. Indeed, it has been reported that younger smokers would substantially increase their probability of attending smoking cessation programmes under assumptions of time and place convenience (Hines, 1996). Younger smokers may also feel overly optimistic about the probability of stopping successfully without any help (Hines, 1996). In contrast, lower confidence in quitting was also associated with this factor. Smokers with low confidence in quitting need more help in order to overcome their deficit in self-efficacy, and citing work and time constraints as a reason for not using support suggests an underestimation of the increased likelihood of success with assisted help, but could also be used as an excuse for not getting help because of a fear of failure (Roddy et al., 2006; Wiltshire, Bancroft, Parry, & Amos, 2003).

Both the notions that smokers should quit on their own and that nothing can help in quitting smoking indicate and confirm an underestimation of the benefits of SSS programmes and that smokers hold misconceptions about them. This corroborates previous findings suggesting a lack of knowledge about the effectiveness of support programs, misunderstandings about the mechanisms of treatment (Christiansen et al., 2015; Hammond et al., 2004; Hughes et al., 2009; Roddy et al., 2006; Smith et al., 2015; van Rossem et al., 2015; Vogt et al., 2010), and scepticism about the need for support (Hammond et al., 2004).

‘Disinterest in quitting’ was less critical as a barrier to attendance in this sample of smokers. An interest in quitting was a criterion for inclusion in the Start2quit study. However, motivation and intentions to quit are not stable attributes in smokers, and this could account for a possible shift in a proportion of our sample expressing disinterest at a later date. Older smokers, more inclined to endorse this factor, might be less likely to accept the evidence that smoking is detrimental to their health (Connolly, 2000), and may also have less faith in the benefits of stopping smoking and the use of pharmacological support (Schofield, 2006).

Also of lesser importance was a lack of social support as a reason for not seeking help. Nevertheless, this factor was more likely to be endorsed by smokers who live in areas of higher deprivation and with other smokers. These social-environmental factors are frequently cited as barriers to successful cessation (Hiscock, Judge, & Bauld, 2011; Challenger, Coleman, & Lewis, 2007; Park, 2004), and this therefore is a group where more support to quit is needed and more targeted promotion is essential.

Overall these results suggest a contrast between the beliefs of older and younger smokers. While older smokers tend to believe that nothing can help and this possibly leads to a disinterest and lack of motivation to quit smoking, other commitments are often put forward by younger smokers as a reason for not getting support to quit. This indicates the need for different targeted strategies for the promotion of services. It also points to similar problems of a lack of sufficient knowledge about the support services, how they operate, and the purpose and rationale of the support. There is a need to challenge the commonly held perception that really wanting to quit is sufficient (Balmford & Borland, 2008; Hughes, 1999), as well as the misperception that receiving formal support will not influence the success of a quit attempt. While services must make themselves convenient, greater assurance of the value of support may encourage more smokers to attend.

This analysis has shown the utility of the TBQ in an English population of smokers interested in quitting smoking following an explicit invitation to attend the SSS. The factors identified in the present study showed good internal consistency and were broadly similar to those identified in the US study (Copeland et al., 2010). The size of the sample, more than adequate to meet the requirement of a factor analysis (de Vet, Adèr, Terwee, & Pouwer, 2005), is a strength of this study. Differences in scoring between the intervention and control groups did not indicate any effect of the additional information received by the intervention group. However, the smokers that participated were recruited for the larger study and were somewhat motivated to quit, and the response rate to the TBQ was low, at <50%. Thus the sample may not be fully representative of the wider population of smokers in England, and ‘disinterest in quitting’, of low importance in this sample, may have been under-represented. It must also be noted that this was an exploratory factor analysis on a new population. Interpretation of factor analysis is subjective and thus substantive conclusions should be drawn with caution.

## 5 Conclusions

The findings of the present study point to the need for tailored recruitment strategies and should be utilised in public health efforts to target promotion and advertising of the services at individual or population level. Use of the TBQ to assess barriers to entering smoking cessation programs will enable the comparison of data across studies and populations, and will allow for the development of strategies to overcome them. ‘Delineating the most common reasons for not seeking treatment could lead to changes to the advertising of services or in information given to smokers by clinicians and could increase treatment seeking’. (Hughes et al., 2009)

## List of abbreviations

IMD: Index of Multiple Deprivation
PCA: Principal Component Analysis
SSS: Stop Smoking Services
TBQ: Treatment Barriers Questionnaire

## Declarations

### Role of funding sources

This work was supported by the National Institute for Health Research HTA Programme (Project Number 08/58/02). The views and opinions expressed herein are those of the authors and do not necessarily reflect those of the HTA Programme, NIHR, NHS or the Department of Health.

### Contributors

HG was the PI, involved in the conception of the study, and contributed to the drafting and revision of the paper. DK conducted the study, analysed and interpreted the data, and wrote the first draft of the paper. SS was involved in the design of the study, and contributed to the drafting and revision of the paper. All authors contributed to critical revision of the manuscript, read and approved the final manuscript and consent to publication in this review.

### Conflict of interest

The authors declare that they have no competing interests.

### Acknowledgments

We would like to thank members of the Start2quit Research Team who managed the recruitment of participants and data collection, also the Local Collaborators and service Managers of the participating Stop Smoking Services, Practice Managers and administrative staff at all of the participating General Practices.

### Ethics approval and consent to participate

The research adhered to the principles outlined in the Declaration of Helsinki and was approved by the London–Surrey Borders Research Ethics Committee. Reference number 10/H0806/20. Participants were informed of the study objectives and provide written informed consent.
